# Automated Measurement of Immature Granulocytes: Performance Characteristics and Utility in Routine Clinical Practice

**DOI:** 10.1155/2012/483670

**Published:** 2012-02-15

**Authors:** Balamurugan Senthilnayagam, Treshul Kumar, Jayapriya Sukumaran, Jeya M., Ramesh Rao K.

**Affiliations:** ^1^Department of Pathology, Chettinad Deemed University, Padur, Kancheepuram 603103, India; ^2^Chettinad Deemed University, Padur, Kancheepuram 603103, India; ^3^Department of Microbiology, Chettinad Deemed University, Padur, Kancheepuram 603103, India

## Abstract

The granulocytic “shift to left” reflects marrow response to bacterial infection, and this may be quantified as band count or immature granulocyte count (IGC). The former value, used widely in neonatal sepsis, has been notoriously difficult to measure accurately and precisely. A reproducible, precise, and accurate counting of immature granulocyte counts may be possible with automation. This study of 200 febrile patients aimed at analysing the performance characteristics of automated immature granulocytes (AIGs) in predicting blood culture and their clinical utility. The absolute (IGC) and relative IG count (IG%) had area under curve (AUC) of 0.69 and 0.66. Moreover, the means of IGC and IG% between culture positive and negative groups were statistically significant suggesting that they are potential markers for bacteremia. IGC of 0.03 × 10^3^ cu·mm and IG% of 0.5% offered sensitivity of 86.3% and 92.2%, respectively, and may be used for screening for bacteremia. Higher values, IGC > 0.3, and IG% > 3 had specificity greater than 90%, although the values were infrequent. It may not be long before that these automated hemograms are put into regular diagnostic use.

## 1. Introduction

Early detection of bacteremia facilitates timely initiation of antimicrobial therapy, reduces morbidity and mortality, and decreases healthcare costs thereby making it a relevant clinical objective. However, there is a considerable timelag before the blood culture results are available for the physician to act upon. This has necessitated studies to address the usefulness of various parameters to predict infection earlier [1]. The manual “band count” used widely in pediatric practice as a marker for bacterial infection has been notoriously difficult to measure accurately and precisely [2, 3]. Therefore, a more reproducible measurement of immature granulocytes might be a useful parameter to predict infection or sepsis. Automated hematology analysers have undergone numerous technical innovations during the last few years. Recent developments permit not only flagging of samples with abnormal cell population but also categorisation and counting of those cells [4]. The Coulter Act Diff 5 counter can perform a 5-part differential leucocyte count and can also enumerate the percentage and absolute number of immature granulocytes (IG% and IGC) using a technology that combines cytochemistry, focused flow impedance, and light absorbance [5, 6]. At this time, automated immature granulocyte measurements are still being evaluated in the research arena and do not form part of routine reporting. Since automated enumeration of immature granulocytes may have better precision, turnaround time, and accuracy, we tried to study the potential of this parameter to predict positive blood culture so that this may find a utility in the routine clinical practice [7–10]. 

## 2. Materials and Methods

 This retrospective observational study included 200 consecutive inpatients suspected to have infection clinically and for whom complete blood count and blood culture have been ordered as part of regular workup. Patients who were currently on antibiotics and those with hematologic malignancy were excluded. Institutional Ethics Clearance (IEC) was obtained prior to starting the study. Informed consent was waived in view of the lack of need for additional blood samples. Complete blood count (CBC) samples were obtained by venipuncture in EDTA vacutainer tubes and analysed on a Coulter Actdiff 5 automated hematology analyser within 4 hours of collection. Multiple hematology parameters including Total WBC Count, 5 part differential (DC), absolute neutrophil count (ANC), and immature granulocyte (IG% and IGC) counts were obtained from the cell counter, and IGC/WBC% and IGC/ANC ratios were computed in the Microsoft Excel Spreadsheet using the primary data. Manual differential counting was performed according to National Committee for Clinical Laboratory Standards (NCCLSs) [11]. The total of promyelocytes, myelocytes, and metamyelocytes was included in the IG count. Blood culture samples were obtained by venipuncture or through indwelling catheters using sterile technique, collecting approximately 1 mL blood into the vials, and vials were incubated in the BACTEC, and samples flagged as positive had aliquots removed for Gram staining and subculture. CBC and blood culture were done using samples obtained at the same time. Statistical software (MedCalc, —Version 11.6.1.0) was utilised for plotting the Receiver Operator Characteristics (ROC) curves, for calculating the areas under curve (AUC) and other statistical measures. Regression analysis was done using Analyse-it software [7].

## 3. Observations and Results

 200 febrile patients during the period of March 2010 to February 2011 for whom complete blood count (CBC) and blood culture were ordered as part of workup were studied. Patients who received prior antibiotics and for whom data were missing were excluded from the study. 102 were adults (>13 years) with a range of 13 to 83 years and a median of 37 years and 98 were children (<12 years), which included 29 neonates (<28 days) and 10 infants (<1 year). The male : female ratio was 0.8. Fifty one of two hundred (25.3%) patients had positive blood culture, of which 27 were adults and 24 children. 17 of 51 (33.3%) grew Gram-positive organisms. The spectrum of organisms isolated is given in Table 1.

**Figure 1:**
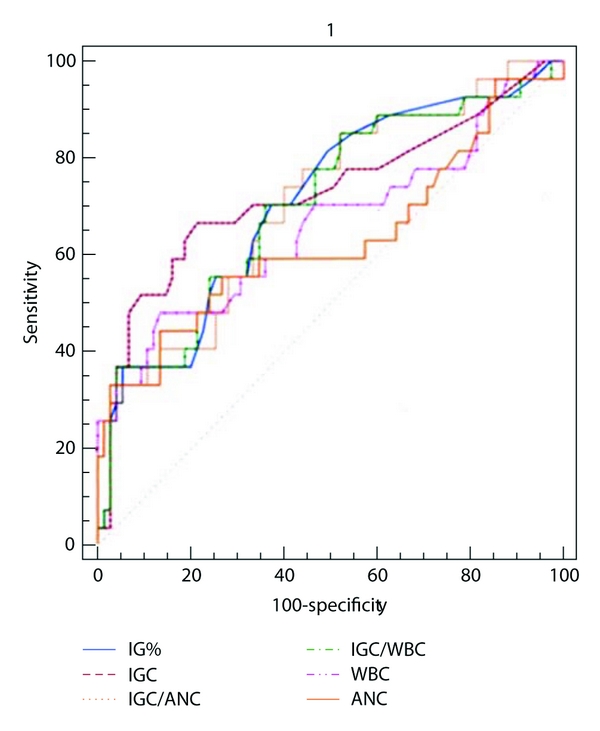
Comparison of ROC curves among the all 6 studied parameters in children.

**Figure 2:**
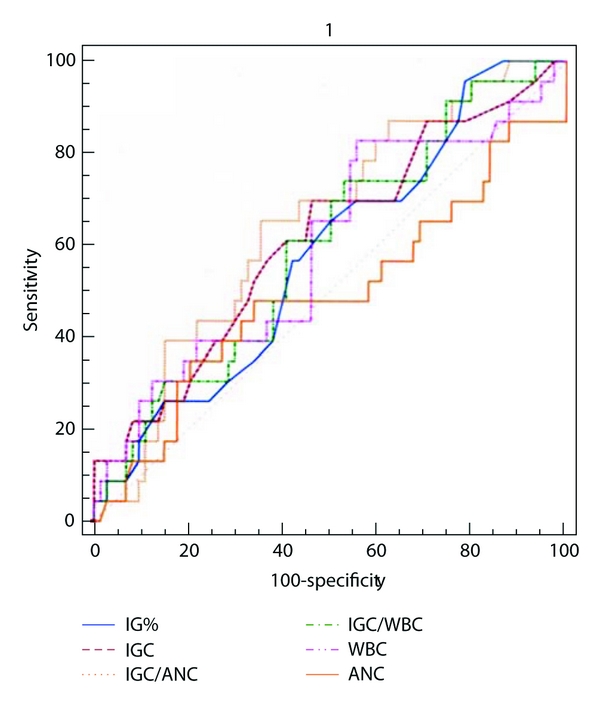
Comparison of ROC curves for all 6 parameters studied in adults.

The range of values and the mean with 95% confidence intervals and standard deviation for IGC, IG%, WBC, ANC, IGC/WBC, and IGC/ANC in adults and children of culture positive and negative subgroups are enumerated in Table 2. Comparison of ROC curves for these six parameters is given in Figures 1 and 2 [12]. area under curve (AUC) (with *z* and *P* values) for this six parameters is given in Table 3. The optimum cut-off values for immature granulocytes were generated from ROC curves. Performance characteristics of IGC and IG% (sensitivity, specificity, positive likelihood ratio, and negative likelihood ratio) at different cut-off values are tabulated in Table 4. This included optimised cutoff of (for IGC and IG% resp.) >0.11 and >1.1; >0.04 and 0.3 in patients under 10 years of age; 0.07 and 0.9 for those over 10 years [13]: >0.03 and >0.5% [14, 15]. The means of 6 parameters between culture positive and negative groups were compared using Student's *t*-test and the results are illustrated in Table 5. 

With in-run imprecision (reproducibility) was performed in 2 different samples of fresh blood run 10 consecutive times [7]. The mean, SD, and coefficient of variation with (CV%) were determined for IGC and IG% and compared with published CV% and SD (Table 6). Passing-Bablock regression analysis [7] was used to compare automated immature granulocyte counts and manual immature granulocyte counts done in 102 random samples, and the results are indicated in Table 7.

## 4. Discussion

 The granulocytic shift to left characterized by the presence of immature granulocytes in the peripheral blood reflects active bone marrow response to bacterial infection. Conventionally, these are classified on the basis of cell morphology by the microscopical examination of a stained blood film into promyelocytes, myelocytes, and metamyelocytes [16, 17]. Technical innovations have permitted automated hematology analysers to identify and count IGs thus offering the possibility of improvements in quality and costs in the laboratory. We evaluated immature granulocytes as measured in Coulter automated analyser as a marker to predict bacterial infection by ROC and AUC analyses.

 The area under curve serves as a single measure that summarises the discriminative ability of a test across the full range of cutoffs, independent of prevalence. ROC plots displayed sensitivity versus 1-specificity, such that areas under the curve (AUC) generated varied from 0.5 to 1.0, with higher values indicating increased discriminatory ability [12]. Confidence intervals on AUCs of ROC plots were calculated using nonparametric assumptions. All 6 parameters studied had AUCs between 0.6 and 0.7 (IGC < IGC/ANC < IG%, IGC/WBC < WBC < ANC) indicating that they are markers of bacteremia. However, ROC curves showed that IGC was a better predictor of infection than WBC and ANC in adults and the ratios IGC/WBC and IGC and ANC did not improve the prediction outcome. In children, IGC and ANC had the similar AUC (0.63) and the ratio IGC/ANC (I/T ratio) offered the maximum AUC (0.65). Among the 51 culture positive cases, 49 had IT ratio >0.65% giving a sensitivity of 96.1%, an observation similar to that of Iddles et al. [17].

 ROC curve is a graphical technique used to assess the test performance at different cut-off levels and for describing and comparing the accuracy of diagnostic tests [12]. The optimal value yielded a sensitivity of 66.67% and specificity of 70.37% for IGC (>0.11) and for IG% (>1.1), 70.37 and 62.67%, respectively. In general, ROC curves showed that all 6 parameters had low sensitivity when high specificity was desired and vice versa. At high values of IGC > 0.3 and IG% > 3, specificity for sepsis was above 90% (93.33% and 94.67%). Although only a small number (20% and 14% for IGC and IG%, resp.) had values above this cutoff, this finding might be used for careful followup.

 The means of IGC (0.62 versus 0.17, *P* < 0.0001) and IG% (3.68 versus 1.46, *P* = 0.0009) in culture positive patients were significantly higher than in culture negative patients. Whereas, in adults, IGC had the most significant difference (*P* < 0.01), WBC and ANC were more significant in children (*P* < 0.001). There were no significant differences between the 34 Gram-positive and 17 Gram-negative cases, and AUCs for all 6 parameters were close to 0.5.

 The biological reference interval for our setup was generated from 100 samples obtained from healthy adults (50 men and 50 women). The reference interval for pediatric population could not be defined owing to practical difficulties. IGC in adults ranged from 0.1 to 0.9 with a mean 0.4 (×10^3^/cu·mm) and IG% from 0.02% to 0.8% with a mean of 0.04%. These observations closely matched with a recent study (2011) by Roehrl et al. [13]. These authors analysed a large outpatient population comprising more than 2400 samples to determine age-stratified normal reference ranges for IGs and recommended the following IG upper reference range limits for routine outpatient use: 0.30%/40.0 *μ*L (−1) (≤10 years) and 0.90%/70.0 *μ*L (−1) (>10 years). These values were higher compared to an earlier study by Sáenz et al. [15] and Bruegel et al. [14] (in healthy blood donors), where the reference absolute counts ranged between zero and 0.03 × 10^3^/cu·mm and the relative counts between zero and 0.4%, without significant differences by gender. This again stresses the need to have an institution-specific reference interval wherever possible. Though our thresholds offered highest specificity, the values recommended by Sáenz et al. [15] and Bruegel et al. [14], that is, IGC >  0.03 × 10^3^/cu·mm and IG% < 0.5% offered highest sensitivity of 86.3% and 92.2%, respectively (82% and 89% in adults; 88% and 96% in children), and since the purpose of these parameters is to screen for and not to diagnose bacteremia, these values are recommended for the purpose of predicting bacterial infection.

 The absolute and relative IG measurements showed excellent reproducibility. With in-run test CVs in our laboratory compared favourably published reproducibility studies [7, 8]. We did not use 3-level commercial controls due to nonavailability. However, these will certainly be an asset for introducing the assay into the clinical laboratory.

 We compared the manual microscopic method and the automated method for IG% and IGC. When the manual count was converted to an absolute count by using the WBC count obtained by the analyser and correlated by using the absolute IG count of the Coulter, the correlation coefficient was 87%. This indicates a strong relationship between the 2 methods of counting IGs and validates the replacement of the traditional manual microscopic IG count by the Coulter. The manual count consistently underestimated the IGs at low counts and is therefore unsuitable and inappropriate as a reference method for counting rare events such as IGs. This is in consonance with findings of other authors [7, 8, 18].

## 5. Conclusion

 Immature granulocyte measurements show promising results in the screening for sepsis and infection. The availability of automated IG count provides for a complete, automated, extended 6-part differential count. Being potential markers for bacterial infection and because no extra cost or additional procedure is involved and therapy may be expedited, IGC and IG% should form part of complete blood count (CBC).

## Figures and Tables

**Table 1:** Spectrum of organisms isolated.

Organism isolated	Number of cases (%) (*n* = 51)
*Streptococcus pneumonia*	2 (4%)
*Staphylococcus aureus*	13 (25%)
*Streptococci sp.*	2 (4%)
*Salmonella sp.*	6 (12%)
*Pseudomonas*	5 (10%)
*Acinetobacter*	3 (6%)
*E. coli*	11 (21%)
*Citrobacter*	4 (8%)
*Klebsiella pneumoniae*	5 (10%)

**Table 2:** Data Characteristics of different parameters in culture positive and negative groups.

	Culture negative (*n* = 75)					Culture positive (*n* = 27)				
	Mean	95% CI	SD	Min	Max	Mean	95% CI	SD	Min	Max
Adults (*n* = 102)										
WBC	8.75	7.62–9.88	4.91	1.4	23.1	14.83	10.67–19	10.52	2.7	39.9
ANC	5.69	4.78–6.61	3.99	0.76	18.32	10.55	6.98−14.12	9.02	0.5	34.33
IGC	0.17	0.058–0.28	0.49	0.01	3.35	0.70	0.19–1.22	1.3	0.02	6.51
IG%	1.58	0.83–2.34	3.29	0.02	25.8	4.86	1.55–8.17	8.38	0.2	38
IGC/WBC%	1.5	0.85–2.1	2.7	0.2	20.6	4.2	1.7–6.2	6.25	0.26	27.4
IGC/ANC%	2.5	1.3–3.6	5.0	0.32	3.9	10	0.91–21.6	28.45	0.59	148

Children (*n* = 98)										
WBC	11.27	9.68–12.85	6.84	1.7	44.9	15.81	10.73−20.89	12.03	3.3	53.8
ANC	4.89	4.10–5.69	3.43	0.17	14.94	7.09	3.89–10.29	7.59	0.68	28.4
IGC	0.17	0.11–0.23	0.25	0.01	1.4	0.53	0.07–0.99	1.08	0.02	4.95
IG%	1.32	1.07–1.58	1.09	0.2	6	2.35	0.47–4.22	4.45	0.5	22.7
IGC/WBC%	1.33	1.08–1.58	1.07	0.13	5.65	2.25	0.73–3.77	3.61	0.39	18.5
IGC/ANC%	3.86	2.85–4.88	4.38	0.25	2.35	11.5	−3.68–26.6	35.85	1.15	179.3

WBC, ANC, and IGC in ×10^3^/cu·mm										

**Table 3:** Area under curve for 6 parameters studied.

Sl. no.	Parameter	All (*n* = 200)					Adults (*n* = 102)					Children (*n* = 98)				
		AUC	SE	95% CI	*z*	*P*	AUC	SE	95% CI	*z*	*P*	AUC	SE	95% CI	*z*	*P*
1	WBC	0.64	0.05	0.54 to 0.73	2.81	0.0025	0.66	0.07	0.56 to 0.75	2.33	0.0195	0.61	0.07	0.47–0.75	1.59	0.0556
2	ANC	0.58	0.05	0.48 to 0.69	1.60	0.0543	0.63	0.07	0.53 to 0.73	1.82	0.0684	0.53	0.08	0.38 to 0.68	0.35	0.3625
3	IGC	0.69	0.047	0.60 to 0.78	4.02	<0.0001	0.73	0.07	0.63 to 0.81	3.53	0.0004	0.63	0.07	0.50 to 0.76	1.9	0.0292
4	IG%	0.66	0.044	0.58 to 0.75	3.68	0.0001	0.71	0.06	0.61 to 0.80	3.52	0.0004	0.60	0.07	0.47 to 0.72	1.44	0.0743
5	IGC/WBC	0.66	0.44	0.58 to 0.75	3.71	0.0001	0.71	0.06	0.61 to 0.79	3.36	0.0008	0.61	0.07	0.51 to 0.71	1.69	0.0913
6	IGC/ANC	0.67	0.04	0.58 to 0.75	3.91	<0.0001	0.71	0.06	0.61 to 0.79	3.52	0.0004	0.65	0.07	0.55 to 0.74	2.4	0.0164

**Table 4:** Performance characteristics at different cut-off values.

	Cutoff	Sensitivity	95% CI	Specificity	95% CI	+LR	−LR
Adults							
IGC							
Optimised	0.11	66.67	46–83.5	78.67	67.7–87.3	3.12	0.42
Roehrl et al.	0.07	70.37	49.8–86.2	57.33	45.4–68.7	1.65	0.52
Bruegel et al.	0.03	81.48	61.9–93.7	33.33	22.9–45.2	1.22	0.56
Institutional	0.1	66.67	46–83.5	78.67	67.7–87.3	3.12	0.42
IG%							
Optimised	1.1	70.37	49.8–86.2	62.67	50.7–73.6	1.88	0.47
Roehrl et al.	0.9	70.37	49.8–86.2	58.67	46.7–69.9	1.7	0.51
Bruegel et al.	0.5	88.89	70.8–97.6	37.33	26.4–49.3	1.42	0.30
Institutional	1	70.37	49.8–86.2	62.67	50.7–73.6	1.88	0.47

Children							
IG%							
Optimised	>0.5	95.83	78.9–99.9	21.62	12.9–32.7	1.22	0.19
Roehrl et al.	0.3	100	85.8–100	31.51	6.7–23.5	1.16	0
IGC							
Optimised	>0.1	70.83	48.9–87.4	54.1	42.1–65.7	1.54	0.54
Roehrl et al.	0.04	87.5	67.6–97.3	21.62	12.9–32.7	1.12	0.58

**Table 5:** Comparison of means (*t*-test) between culture positive and culture negative groups.

All						
	WBC	ANC	IGC	IG%	IGC/WBC%	IGC/ANC%
Difference	5.29	3.62	0.45	2.23	1.85	8.71
SE	1.24	0.87	0.11	0.66	0.51	2.68
95% CI	2.84–7.74	1.91–5.33	0.23–0.67	0.93–3.53	0.84–2.86	3.43–13.99
*t*	4.25	4.18	4.04	3.40	3.61	3.25
DF	198	198	198	198	198	198
	<0.0001	<0.0001	0.0001	0.0008	0.004	0.0013

**Table 6:** Within-run reproducibility (imprecision) performed on fresh blood samples.

	Mean	SD	CV%	Published SD and CV [7]
Sample 1				
IGC (×10^3^/cu·mm)	0.38	0.0265	6.96	SD < 0.12, CV < 25%
IG%	3.57	0.32	9.01	SD < 1.5, CV < 25%

Sample 2				
IGC (×10^3^/cu·mm)	7.87	0.0058	7.87	SD < 0.12, CV < 25%
IG%	1.1	0.1	9.09	SD < 1.5, CV < 25%

**Table 7:** Estimate of agreement between different counting methods based on Passing-Bablock regression analysis for immature granulocytes.

		Estimate	95% CI
Automated IG% versus Manual IG%	Slope	0.94	0.83–1
	Intercept	−0.38	−0.40–0.25

Automated IGC versus Manual IGC	Slope	0.93	0.86–1
	Intercept	−0.02	−0.03–(−0.02)
